# Scheduling and Power Control for Wireless Multicast Systems via Deep Reinforcement Learning †

**DOI:** 10.3390/e23121555

**Published:** 2021-11-23

**Authors:** Ramkumar Raghu, Mahadesh Panju, Vaneet Aggarwal, Vinod Sharma

**Affiliations:** 1 Indian Institute of Science, Karnataka 560012, India; ramkumar@iisc.ac.in (R.R.); mahadesh@iisc.ac.in (M.P.); vinod@iisc.ac.in (V.S.); 2 School of Industrial Engineering and School of Electrical and Computer Engineering, Purdue University, West Lafayette, IN 47907, USA

**Keywords:** multicasting, scheduling, queuing, deep reinforcement learning, quality of service, power control, dynamics tracking, multi-timescale stochastic optimization

## Abstract

Multicasting in wireless systems is a natural way to exploit the redundancy in user requests in a content centric network. Power control and optimal scheduling can significantly improve the wireless multicast network’s performance under fading. However, the model-based approaches for power control and scheduling studied earlier are not scalable to large state spaces or changing system dynamics. In this paper, we use deep reinforcement learning, where we use function approximation of the Q-function via a deep neural network to obtain a power control policy that matches the optimal policy for a small network. We show that power control policy can be learned for reasonably large systems via this approach. Further, we use multi-timescale stochastic optimization to maintain the average power constraint. We demonstrate that a slight modification of the learning algorithm allows tracking of time varying system statistics. Finally, we extend the multi-time scale approach to simultaneously learn the optimal queuing strategy along with power control. We demonstrate the scalability, tracking and cross-layer optimization capabilities of our algorithms via simulations. The proposed multi-time scale approach can be used in general large state-space dynamical systems with multiple objectives and constraints, and may be of independent interest.

## 1. Introduction

Content services, such as Netflix, Prime Video, etc., have dramatically increased the demand for high-definition videos over mobile networks. Almost 78% of mobile data traffic is expected to be due to these mobile videos [1]. It is observed that the request traffic for these contents have multiple redundant requests [2]. Next generation wireless networks are being constantly upgraded to satisfy these exploding demands by exploiting the nature of the request traffic. Serving the redundant requests simultaneously is a natural way to utilize network resources efficiently. Thus, efficient multicasting is studied widely in the wireless networking community.

A multicast queue with network coding is studied in [3] with an infinite library of files. The case of slotted broadcast systems with one server transmitting to multiple users is studied in [4]. Some recent works [5] use coded caching to achieve multicast. This approach uses local information in the user caches to decode the coded transmission and provides improvement in throughput by increasing the effective number of files transferred per transmission. This throughput may get reduced in a practical scenario, due to queuing delays at the base station/server. Ref. [6] addresses these issues, analyzes the queuing delays and compares it with an alternate coded scheme with LRU caches (CDLS), which provide improvement over the coded schemes in [5]. A more recent work in this direction, Ref. [7] provides alternate multicast schemes and analyzes queuing delays for such multicast systems. In [7], it is shown that a simple multicast scheme can have significant gains over the schemes in [5,6] in a high-traffic regime.

We further study the multicast scheme proposed in [7] in this paper. This multicast queue merges the requests for a given file from different users, arriving during the waiting time of the initial requests. The merged requests are then served simultaneously. The gains achieved by this simple multicast scheme, however, are quickly lost in wireless channels, due to fading. It suffers from users with bad channels, thereby decreasing the QoS, even for users with good channels. In [7], we studied this problem and proposed novel schemes, which provide significant multicast gains under fading as compared to the simple multicast. In [7], we also theoretically analyzed these queuing schemes, showed that our queues are always stable and provided approximate theoretical expressions for mean sojourn times. Further, in [8], we showed that state-dependent power control under an average power constraint can significantly improve the average delays experienced by users.

The queuing schemes and the power control policy proposed in [8], though they provide improved delays, have the following limitations. (1) The queuing scheme which performs best depends on the system parameters, such as the size of the system, the request rate, etc. (2) The algorithm to obtain the power control policy is not scalable with the number of users and the number of states of the channel gains. Additionally, the policy does not adapt to changing the system statistics, which in turn, depends on the power control policy. (3) The queuing schemes and power control are dealt with individually. This paper tries to overcome the above limitations of the scheme in [8].

We first provide algorithms for the two optimization problems individually and then combine the two algorithms to obtain the overall optimal queuing strategy and the power control. Stochastic optimization ([9]) is a useful tool to obtain the optimally parameterized queuing strategy. However, for the convergence of stochastic optimization algorithms, a careful approximation of stochastic gradients is necessary. One challenge here is that the cost to be optimized is the mean stationary sojourn time of the requests to be delivered. We propose a new deep assisted gradient approximation algorithm, where the novelty is in deriving the gradients from a deep network assisted by a memory. This memory helps retain the history of the explored regions and also allows adaptation to changing system dynamics in an online fashion. The replay memory and online training of the deep network adds an important feature called importance sampling to the stochastic optimization, which improves the confidence (lower variance) in the gradient descent steps.

Multicast systems with power control can be conveniently modeled as a Markov decision process (MDP) but with large state and action spaces. Obtaining transition probabilities and the optimal policy, however, for such large MDPs is not feasible. Reinforcement learning, particularly deep reinforcement learning [10], is a natural tool to address such problems. Reinforcement learning can be used even when the transition probabilities are not available. However, a large state/action space can still be an issue. Using function approximation via deep neural networks can provide significant gains. Several deep reinforcement learning techniques, such as deep Q-network [11], trust region policy optimization (TRPO) [12], proximal policy gradient (PPO) [13], etc., have been successfully applied to several large state-space dynamical systems, such as Atari [14], AlphaGo [15], etc. DQN is based on value iteration. TRPO and PPO are policy-gradient-based methods. Policy–gradient methods often suffer from high variance in sample estimates and poor sample efficiency [10]. Value-iteration-based deep RL methods, such as DQN, have been theoretically shown to have better performance [16], due to target network and replay memory and providing a global minimum.

We propose a constrained optimization variant of DQN based on multi-timescale stochastic gradient descent [9] for power control, which can track the system statistics. Finally, we develop an algorithm which combines the above two algorithms to obtain an optimal queuing strategy and power control policy.

The major contributions of this paper are as follows:

- A novel deep assisted stochastic gradient descent (DSGD) algorithm for obtaining the best queuing strategy from a given set.
- Proposing two modifications to DQN to accommodate constraints and system adaptations. The constraints can be met by using a Lagrange multiplier. The appropriate Lagrange multiplier is also learned via a two-timescale stochastic gradient descent. We call this algorithm adaptive constrained DQN (AC-DQN).
- Unlike DQN, AC-DQN can be applied to the multicast systems with constraints, as in [8], to learn the power control policy, online. The proposed method meets the average power constraint while achieving the global optima as achieved by the static policy proposed in [8] for a small-scale setup of the problem.
- We demonstrate the scalability of our algorithms with the system size (number of users, arrival rate, complex fading).
- Finally, using the above two algorithms, we propose a generalized algorithm called integrated DSGD and AC-DQN (IDA) to optimize systems with multiple objectives and constraints. Particularly, this algorithm is useful in any wireless network with cross-layer objectives, such as ours. IDA is a three-timescale stochastic optimization algorithm for obtaining both the queuing strategy (unconstrained network layer objective) and power control (constrained physical layer objective), simultaneously.
- We also show that AC-DQN and IDA can track the changes in the dynamics of a non-stationary system, e.g., change of arrival rate or number of users over the time of a day, and achieve optimal performance.

We show via simulations that our algorithms choose the optimal policy among the given set of policies. Additionally, the power control policy obtained via our algorithm improves the delay performance of the multicast network by more than 50%, compared to the constant power policy. Our algorithms work equally well when we replace DQN with its improvements, such as DDQN [17]. In fact we ran our simulations with the DDQN variant of AC-DQN and achieved similar performance. It is worth noting that, even though we demonstrate the power of deep (reinforcement) learning, in improving schemes in [7,8], the proposed deep algorithms themselves are generic and can be applied to any dynamical system with multiple objectives, constraints and large state spaces.

### Related Works

**Queuing and Power control in Multicast Systems:** Multicast queuing and scheduling is studied in [3,18,19,20]. The works in [3,18,19] propose schemes for network-coded multicast systems and analyze the stability of the proposed multicast queues. Unlike these works, we use, as in our previous work [7,8], a simple uncoded multicast queue, which is always stable. In [7], we show that our queuing schemes perform much better than the coded multicast schemes in high traffic regimes. In the current work, we improve the results in [7,8] by providing novel deep-learning-based queuing strategies. Ref. [20] proposes a multicast scheduling scheme for Poisson traffic. However, there is no power control, and the proposed queue is not always stable. The effect of multicasting and caching on energy cost in a delay-tolerant content-centric network is studied in [21]. The work, however, does not consider the effect of the queuing delay considered in the paper, and does not have any constraints on the transmit power. Power control in multicast systems is studied in [22,23,24]. In [22], power allocation optimizes the ergodic capacity while maintaining certain minimum rate requirements at the users and average power constraints. In [23], the authors minimize a utility function via linear programming under SINR constraints at the users and transmit power constraints at the transmitter. Refs. [22,23] derive an optimal power control policy for delivery to *all* the users, whereas this paper considers delivery to a random subset of users requesting a file at that time. Additionally, the power control policies in [22,23] require knowledge of system statistics and are not *scalable* for our system. Ref. [24] considers MDP-based scheduling and power control in content-centric multicast systems. The work in [24] uses fixed channel states, requires statistics of queue state transitions and does not have any constraint on the average transmit power. Further, the state dimension of the system increases with both the number of users and files, whereas in our work, the dependence is only on the number of users. Thus, compared to the above-mentioned works, our scheme is more practical, computationally scalable, does not require knowledge of system statistics (traffic intensity, and fading distributions) and can track changing system statistics.

**Deep Learning in Wireless Multicast systems:** The ability of DeepRL to handle large state-space dynamic systems is being exploited in various multicast wireless systems/networks. In [25], the authors study a resource allocation problem in unicast and broadcast transmissions. The DeepRL agent learns and selects the power and frequency for each channel to improve the rate under some latency constraints. Like in our work, they also introduce constraints via Lagrange multipliers. However, the Lagrange multiplier is constant, and the agent does not learn it. Thus, the agent also does not adapt if the system dynamics changes, as the Lagrange constant is fixed and the learning rate decays with time. To obtain the appropriate Lagrange multiplier is computationally expensive and requires known system statistics. Another work, Ref. [26], applies unconstrained deep reinforcement learning to multiple transmitters for a proportionally fair scheduling policy by adjusting individual transmit powers. Ref. [27] applies DeepRL in queuing in a coded caching-based multicast system, which is shown to be inferior to our multicast schemes in high traffic rate regions. For more literature on deep learning applications to wireless multicast systems, see the detailed survey in [28].

For a detailed exposition on constrained MDPs, see [29]. Some recent works on reinforcement learning provide convergence guarantees for the tabular model-free Q-learning, using the minimax approach [30], model-based online policy optimization approach [31], tabular model-based Q-learning approach [32], and tabular primal-dual approach [33]. The approaches in these works are not demonstrated on large state spaces, in part due to the increased complexity of the tabular algorithm in [30,32,33], and linear MDP assumption in [31]. Ref. [34] introduces constrained reinforcement learning based on TRPO. Unlike ours, the work considers discounted constraints. In [35], a Lagrange-based actor–critic approach for constrained RL is proposed. Since [31,34,35] are policy-based approaches, they suffer from high variance when multiple evaluations are unfeasible. In [36], an alternate approach with two value functions for reward and constraint (cost) with an actor–critic policy update is proposed. Here, at each step, a convex relaxation-based optimization is used to obtain the optimal parameter of value functions. We note that the convex optimization step at each iteration is computationally more intensive than a simple SGD step. Thus, the above-mentioned policy iteration methods either have high variance in practical systems or are computationally intensive. These issues make it difficult to track the changing dynamics in practical systems, as we can in our case. To the best of our knowledge, ours is the first constrained value iteration-based deep RL algorithm for constrained MDPs. The use of replay memory and a target network helps reduce the estimator variance in our algorithm. These features also increase the practical applicability of our algorithm.

The rest of the paper is organized as follows. Section 2 explains the system model and motivates the problem. Section 3 presents our deep-learning-based optimal queuing algorithm. Section 4 motivates the power control problem and briefly explains the power control algorithm proposed in [8]. Section 5 presents the proposed DeepRL algorithm AC-DQN for scalable, improved power control. Section 6 presents our novel deep multi-timescale algorithm to achieve scalable cross-layer optimization of queuing and power control and provides optimal performance for the multicast system. Section 7 demonstrates our algorithms via simulations, and Section 8 concludes the paper.

## 2. System Model

**Figure 1:**
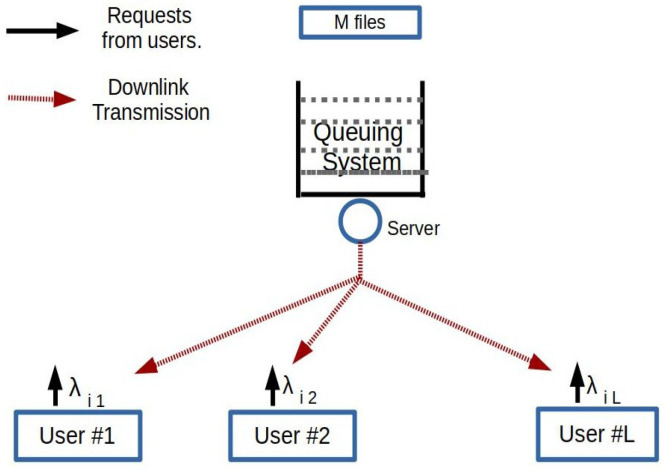
System model.

We consider a system with one server transmitting files from a fixed finite library to a set of users (Figure 1). We denote the set of users by L={1,2,⋯,L} and the set of files by M={1,2,⋯,M}. The request process for file *i* from user *j* is a Poisson process of rate $\lambda_{ij}$, which is independent of the request processes of other files from user *j* and also from other users. The total arrival rate is $\lambda =\sum_{i,j}\lambda_{ij}$. The requests of a file from each user are queued at the server until the user successfully receives the file. All the files are of length *F* bits. The server transmits at a fixed rate, *R* bits/s. Thus, the transmission time for each file is T=F/R.

The channels between the server and the users experience time varying fading. The channel gain of each user is assumed to be constant during the transmission of a file. The channel gain for the *j*th user at the *t*th transmission is represented by $H_{j}\left(t\right)$. Each $H_{j}\left(t\right)$ takes values in a finite set and forms an independent identically distributed (i.i.d) sequence in time, as in [37]. The channel gains of different users are independent of each other and may have different distributions. Let $H=(H_{1},\cdots ,H_{L})$.

Since the requests from the users are queued at the server, every request awaits its turn for transmission, and thus experiences a queuing delay, which is random in nature. The distribution of this random delay depends on the queuing policy. Additionally, unsuccessful transmissions due to fading adds further delay, experienced by each request. We denote by random variable *D* the overall delay experienced by each request due to both queuing and fading. If $t_{A}$ is the time of arrival of a request to the server and $t_{S}$ is the time instance representing the end of successful transmission/service of the request, then the random delay/sojourn time *D* is given by $D=t_{S}-t_{R}$. Further, E[D] denotes the stationary mean sojourn time experienced by each request.

More details of the system are described in the following sections as follows. Section 2.1 describes the basic multicast queue proposed in [7]. The queuing schemes to mitigate the effects of fading studied in [8] are also presented. Section 2.2 parameterizes the queuing schemes. Section 3 provides an online learning scheme to obtain the optimal policy for a given setup. In Section 4.1 and Section 4.2, we summarize the results from [8], which show that using power control can further improve the performance and the algorithm used to obtain the optimal power policy. We will see that this algorithm is not scalable. Then in Section 4.3, we provide the MDP of the power control problem. In Section 5, we present the scalable DeepRL solution for this formulation.

### 2.1. Multicast Queue

For scheduling transmissions at the server, we consider the *multicast queue* studied in [8]. In this system, the requests for different files from different users are queued in a single queue, called the multicast queue. In this queue, the requests for file *i* from all users are merged and considered a single request. The requested file and the users requesting it is denoted by $(i,L_{i}$). In other words, $L_{i}$ is the list of users interested in file *i*. A new request for file *i* from user *j* is merged with the corresponding entry $L_{i}$ if it already exists. Otherwise, it is appended to the tail of the queue. The service/transmission of file *i* serves all the users in $L_{i}$, possibly with errors, due to channel fading.

The random subset of users served by the multicast queue at the *t*th transmission is denoted by the random binary vector, $V\left(t\right)=(V_{1}\left(t\right),\cdots ,V_{L}\left(t\right))$, where $V_{j}\left(t\right)=1$ implies that the user *j* has requested the file being transmitted; otherwise, $V_{j}\left(t\right)=0$. From (Theorem 1, [7]), V(t) has a unique stationary distribution.

It is shown in [7] that the above multicast queue performs much better than the multicast queues proposed in the literature before. *The main difference compared to previous multicast schemes is that in this scheme, all requests of all the users for a given file are merged together over time. One direct consequence of this is that the queue length at the base station does not exceed M. Thus, the delay is bounded for all traffic rates. It is worth noting that this is unique to our queues in [7] and none of the queues proposed in the literature have this feature. In fact, the mean delays are often better than the coded caching schemes proposed in the literature as well for most of the traffic conditions.*

In a fading scenario, where the different users have independent fading, the performance of this scheme can significantly deteriorate because of the multiple retransmissions required to successfully transmit to all the users needed. Thus, in [8], multiple queuing strategies are proposed and compared to recover the performance of the system and reduce the mean delay substantially. Some of these are also fair to different users in the sense that the users with good channel gains do not suffer because of users with bad channel gains. We comment more on this in the following. We now briefly present the schemes proposed in [7,8] for clarity.

**Retransmit:** This is the simplest scheme proposed in [7]. Here, the multicast queue is serviced from head to tail. The head of the line is retransmitted until all the users in it are serviced. The new requests are added to the queue in a similar manner to the simple multicast. This naive scheme works very well in a low request rate regime; however, it performs poorly for high request rates and severely deteriorates the delays experienced by users with good channels.

**Single queue with loop-back (1-LB):** The multicast queue is serviced from head to tail. When a file is transmitted, some of the users receive the file successfully and some users may receive the file with errors. In the case of unsuccessful reception by some users, the file is retransmitted. A maximum of *N*(1≤N≤∞) transmission attempts are made. If there are some users who have not received the file within *N* transmission attempts, the request (tuple $\left(i,L_{i}\right)$ with $L_{i}$, now modified to contain only the set of users who have not received the file *i* successfully) is fed back to the queue. If there is another pending request in the queue for the same file (a request for the file which came during the current transmission), it is merged with the existing request. Otherwise, a new request for the same file with unsuccessful users is inserted at the tail of the queue.

**Defer queue with loop back (2-LB):** This strategy has two queues for servicing the requests: a multicast queue and a defer queue. The multicast queue is similar to the queue mentioned in the beginning of this section and is serviced from head to tail. The defer queue is an additional queue to handle unsuccessful transmissions as follows. When a file is transmitted, some users may receive the file with errors. In the case of unsuccessful reception by some users after a maximum of *N* transmissions, the file request and the unserviced users are queued in the defer queue. Such requests stay in the defer queue until a new request for the same file arrives. On the arrival of the new request, the new request is merged with the older requests in the defer queue and moved to the tail of the multicast queue. If no such old requests exist in the defer queue, the new request is merged/added to the multicast queue. This queue is shown to provide lower delay to good channel users than to bad channel users.

The performance of each of these queues depends on the system parameters, transmission power policy, arrival rate, etc. If the channel gain statistics of different users are different, say, one group with good statistics and another with bad statistics, then the rate of transmission *R* and *N* can decide on the preference one is giving to the two groups of users. A higher *R* and lower *N* will give more preference to the good users at the cost of the bad users. For simplicity of presentation, we consider the case of N=1 for all the queuing strategies in this paper.

### 2.2. Parametrization of Queueing Strategies

To adaptively optimize the queuing strategy according to the system parameters, it is convenient to first parameterize them. We propose a simple parametrization, using probabilities for each queuing strategy. That is, at the end of every service instance, if some users have not received the file successfully, the multicast queue chooses to retransmit the head of the line (HoL) request with probability $p_{1}$, loopback HoL with probability $p_{2}$, or defer HoL with probability $p_{3}$, such that $\sum_{j=1}^{j=3}p_{j}=1$. Thus, $\bar{p}=\left[p_{1},p_{2},p_{3}\right]$ parameterizes the queuing strategy. Here, $\bar{p}\in P$, where P is the probability simplex, $P=\{\left[p_{1},p_{2},p_{3}\right]\in {\left[0,1\right]}^{3}:\sum_{j=1}^{j=3}p_{j}=1\}$. Observe that $\bar{p}=\left[1,0,0\right],\;\left[0,1,0\right],$ and [0,0,1] represent retransmit, loopback, and defer strategies. In the next section, we provide an algorithm to obtain the optimal $\bar{p}$.

## 3. Deep Learning for Optimal Queueing

We are interested in finding the optimal $\bar{p}$ among the parameterized queuing strategies in Section 2.2 that gives the least average delay. From our previous work (Proposition 1, [7]), it can be shown that for any parameter $\bar{p}$, there exists a stationary mean sojourn time, $E_{\bar{p}}\left[D\right]$, where *D* is the sojourn time and *E* is the expectation. In this section, we propose an online deep learning algorithm to learn ${\bar{p}}^{*}=\underset{\bar{p}\in P}{argmin}E_{\bar{p}}\left[D\right]$. However, the map $f:\bar{p}\mapsto E_{\bar{p}}\left[D\right]$ is quite complex, and it is very difficult to obtain its closed-form expression.

Since we do not have a closed-form expression, we depend on noisy observations of *f*, the mean sojourn time, from the system to obtain the optimal strategy, ${\bar{p}}^{*}$. Here is where the deep neural network (DNN) fits in. They are state-of-the-art tools used for several learning problems, especially regression. Before we proceed with the motivation for using DNN, it is worth mentioning that several stochastic approximation algorithms, such as simultaneous perturbation stochastic approximation ([38], pp. 41–76), exist for such noisy function optimization. However, the convergence of such algorithms is prone to high variance in the gradient estimate and often leads to suboptimal results. In fact, we tried SF-SPSA ([38], pp. 77–102) in our system and observed that the algorithm leads to a suboptimal point in many cases. ReLU (rectified linear unit) based deep neural networks (DNN), on the other hand, are adept at approximating such complex functions on compact subsets, such as P [39]. Particularly, it is seen that DNN can provide better generalization in function approximation, even with noisy training data [40]. Further, DNNs are also known to provide good gradient approximations for the approximated function [41]. This motivates us to use DNN to approximate $f(\bar{p})$ as $f_{\theta }\left(\bar{p}\right)$, where θ is the weight parameter of the DNN. Further, the gradients required for optimization are derived using the finite difference method on $f_{\theta }\left(\bar{p}\right)$. Another important feature of our algorithm is the replay memory. This idea is borrowed from the reinforcement learning setting [42]. It helps us in storing previously seen noisy function observations and using it for training the DNN in online fashion.

The replay memory and online training of the DNN are the important features of our algorithm. Online training inherently adds an importance sampling [43] feature to our algorithm, that is, we train our neural network only with samples that are more informative. This is shown to accelerate the DNN training time [43]. We see in our algorithm that this happens naturally, as training samples for the neural network come from the parameter $\bar{p}$ update step. These samples give more information about the neighborhood of the point that the algorithm is currently in, thereby improving the confidence/variance in the descent direction. We now present our algorithm, deep assisted stochastic gradient descent, for obtaining the optimal queuing strategy.

### Deep Assisted Stochastic Gradient Descent (DSGD)

Our algorithm has three steps as follows:

- Obtaining noisy observation $\hat{f}$ of the function *f* at random points and storing it in replay memory, $M_{D}$. This provides us with the initial training set.
  To obtain $\hat{f}$ for a randomly generated point $\bar{p}$, the system is set to follow policy $\bar{p}$ and run until the $S_{approx}$ services are completed. Let $d_{i}$ be the sojourn time of the *i*th successfully served request in $S_{approx}$ services. These are stored in a temporary memory $\bar{D}$. From $d_{i},i\in \left[\right|\bar{D}\left|\right]$ compute the following:
  (1) $$\hat{f}=\frac{1}{|\bar{D}|}\sum_{i=1}^{|\bar{D}|}d_{i}$$
  The point $\left(\bar{p},\hat{f}\right)$ is stored in $M_{D}$, and $\bar{D}$ is cleared.
- Sample a minibatch of points from $M_{D}$, and uniformly randomly and train $f_{\theta }$ as follows:
  (2) $$\theta \leftarrow \theta -\eta_{1}\nabla_{\theta }L_{f_{\theta }}$$
  where $L_{f_{\theta }}$ is the mean square error obtained from the minibatch sampled from the replay memory, given by $L_{f_{\theta }}=\sum_{i=1}^{n}{\left(f_{\theta }\left({\bar{p}}_{i}\right)-{\hat{f}}_{i}\right)}^{2}/n$.
- Obtain the numerical gradient of $f_{\theta }$ at the last executed point $\bar{p}$ and perform a gradient descent as follows:
  (3) $$\bar{p}\leftarrow P\left(\bar{p}-\eta_{2}\nabla_{\bar{p}}f_{\theta }\left(\bar{p}\right)\right)$$
  Obtain the noisy observation of *f* at the new point. Store the new $\left(\bar{p},\hat{f}\right)$ to the replay memory, $M_{D}$. P is the projection operator that projects the input to the probability simplex as follows:
  (4) $$P\left[r_{1},r_{2},r_{3}\right]={\left\{\left[r_{1},r_{2},r_{3}\right]\right\}}^{+}/\sum_{i=1}^{3}{\left\{r_{i}\right\}}^{+})$$
  where the element wise operator ${\left\{\cdot \right\}}^{+}=max\left\{0,\cdot \right\}$, and $r_{i}\in R,\;i=1,2,3$.
- $\eta_{1}$ and $\eta_{2}$ are learning parameters and must follow the learning rate relationships of the multi-timescale stochastic gradient descent [9] given in (17) in Section 5. The detailed algorithm is given in Algorithm 1.

Note 1: The initial training phase $\left(t<T_{train}\right)$ and the explorative noise $Unif({\left[0,\epsilon_{t}\right]}^{3})$ in Algorithm 1 avoid pathological zero gradients, which may stall the algorithm prematurely. Further, we use the Adam optimizer [44] in all our SGD steps for gradient annealing.

Note 2: The online learning of the DNN weights is important for adapting to a changing environment, which is experienced in practical systems. The samples in replay memory $M_{D}$ are collected along the descent trajectory in small steps. Thus, DNN is trained to learn the local surface/neighborhood at each step and improve the gradients as the algorithm progresses. This is importance sampling for DNN.

Section 7.2 provides the simulation results of DSGD for a multicast system with constant transmit power.
**Algorithm 1** Deep assisted stochastic gradient descent (DSGD) algorithm
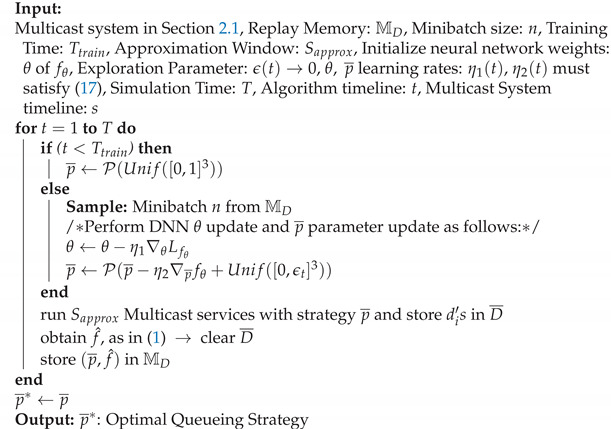


## 4. Power Control for Multicast Queue

We now proceed to describe the power control in the Multicast setup. Adapting the transmit power based on the system and environment state under certain system constraints helps in providing the power control that may improve QoS, which is quantified by the mean user delay under stationarity. It is shown in [8] that by choosing the transmit power based on the channel gains, the system performance improves. We describe the system constraint, a power control model and the MADS power control algorithm proposed in [8] in this section. We then end this section with the Markov decision process formulation of the entire system that aids in the development of the deep reinforcement learning based power control algorithm.

### 4.1. Average Power Constraint

Depending on the value of H(t) and V(t) at time *t*, the server chooses transmit power $P_{t}$ based on a power control policy $P_{t}=\pi \left(H\left(t\right),V\left(t\right)\right)$. Choosing a good power control policy is the topic of this section. The state $S_{t}$ of the system at time *t* is (H(t),V(t)). Let $\;P_{S_{t}}$ be the power chosen by a policy for state $S_{t}$ and let $R(S_{t},P_{S_{t}})$ be the number of successful transmissions for the selected power $P_{S_{t}}$ during the *t*th service.

For a fixed transmission rate *C* and for a given channel gain H(t) of users, the transmit power requirement $P_{req}$ (from Shannon’s Formula) for user *j* is (assuming file length is long enough) the following:

(5) $$P_{req}\left(j,S_{t}\right)=\frac{N_{g}}{H_{j}^{2}\left(t\right)}\left(2^{C/B}-1\right),$$

where *B* is the bandwidth and $N_{g}$ is the Gaussian noise power at receiver *j*. Here, for simplicity, we take the ideal Shannon formula in (5), which can be easily modified to make it more realistic ([45], Chapter 14). Thus, the reward for the chosen power control policy, during the *t*th transmission, is given by the following:

(6) $$R\left(S_{t},P_{S_{t}}\right)=\sum_{j=1}^{L}V_{j,S_{t}}\;1_{\left\{P_{S_{t}}>P_{req}\left(j,S_{t}\right)\right\}}\left(t\right),$$

where $V_{j,S_{t}}=1$ if the user *j* has requested the file in service, and $V_{j,S_{t}}=0$ otherwise. We now describe the mesh adaptive direct search (MADS) power control policy.

### 4.2. MADS Power Control Policy

The power control policy in [8] is derived from the following optimization problem,

(7) $$\max_{\left\{P_{1},\cdots ,P_{K}\right\}}\sum_{k=1}^{K}q_{k}R_{k}\;s.t.\;\sum_{k=1}^{K}q_{k}P_{k}\leq \bar{P}\;\mathrm{and}\;P_{k}\geq 0,k\in \left[K\right],$$

where [K]{1,⋯,K}, $\bar{P}$ is the average power constraint, *K* is the total number of states, $P_{k}$ is the power chosen by the policy in state *k*, $q_{k}$ is the stationary distribution of state k∈[K] and are assumed to be known apriori, and $R_{k}$ is the reward for state *k*, given as $R_{k}=R\left(S_{t}=k,P_{t}=P_{k}\right)$. This is a non-convex optimization problem since the reward in Equation (6) is a simple function (linear combination of indicators). Mesh adaptive direct search (MADS) [46] is used in [8] to solve this constrained optimization problem and obtain the power control policy. Though MADS achieves a global optimum, it is not scalable, as its computational complexity is very high.

The state space and action space of this problem can be very high, even for a moderate number of users and channel gains, e.g., a system with *L* users and *G* channel gain states has $O(2^{L}G^{L})$ states. Therefore, in this paper, we propose a deep reinforcement learning framework. This not only provides optimal solution for a reasonably large system, but does so without knowing the arrival rates and channel gain statistics. In addition, we show via simulations that we can track an optimal solution, even when the arrival and channel gain statistics change with time.

### 4.3. MDP Formulation

The above system can be formulated into a finite state, action Markov decision process denoted by a tuple (S,A,r,P,γ—the state space, action space, reward, transition probability, and discount factor), where the transition probability $P\left(S_{t+1}|S_{0},P_{0},...,S_{t},P_{t}\right)=P\left(S_{t+1}|S_{t},P_{t}\right)$, policy π chooses power $P_{t}∼\pi \left(.|S_{t}\right)$ in state $S_{t}$ and the instantaneous reward $r_{t}=R\left(S_{t},P_{t}\right)$.

The action–value function [47] for this discounted MDP for policy π is as follows:

(8) $$\begin{array}{cc} Q^{\pi } & \left(s,a\right)=E\left[\sum_{t=0}^{\infty }\gamma^{t}r_{t}|S_{0}=s,P_{0}=a\right]. \end{array}$$

where 0<γ<1. The optimal Q-function, $Q^{*}$, is given by $Q^{*}\left(s,a\right)=\max_{\pi }\;Q^{\pi }\left(s,a\right)$ and satisfies the following optimality relation:

(9) $$Q^{*}\left(s,a\right)=r\left(s,a\right)+\max_{a^{\prime }}\;\gamma E\left[Q^{*}\left(s^{\prime },a^{\prime }\right)\right],$$

where $s^{\prime }$ is sampled with distribution P(.|s,a). If we know the optimal Q-function $\left(Q^{*}\right)$, we can compute the optimal policy via $\pi \left(s\right)=\underset{a^{\prime }}{\arg\max}\;Q^{*}\left(s,a\right)$. We know the transition matrix of this system and hence, can compute the *Q*-function. However, the state space is very large, even for a small number of users, rendering the computations unfeasible. Thus, we use a parametric function approximation of the *Q*-function via deep neural networks and use DeepRL algorithms to obtain the optimal $Q^{*}$. Our cost function is the stationary mean sojourn time. To obtain a policy which minimizes this, we actually should be working with the average cost MDP instead of discounted MDP. However, the RL formulation for this problem is defined for the discounted case, the average case being more complicated. If we take the discount factor gamma close enough to 1, then the optimal policy obtained via the discounted problem is often close to the average case problem.

Further, to introduce the average power constraint in the MDP formulation, we look at the policies achieving the following:

(10) $$Q^{*}\left(s,a\right)=\max_{\pi :C_{P}\leq \bar{P}}\;Q^{\pi }\left(s,a\right)$$

where

(11) $$C_{P}=E\left[\lim_{T\to \infty }\frac{\sum_{t=0}^{T}P_{t}}{T}\right]$$

is the long term average power. We use the Lagrange method for constrained MDPs [29] to achieve the optimal policy. In this method, the instantaneous reward is modified as follows:

(12) $$r_{t}=R\left(S_{t},P_{t}\right)-\beta P_{t},$$

where β is the Lagrange constant achieving optimal $Q^{*}$ while maintaining $C_{P}\leq \bar{P}$. Choosing β wrongly provides the optimal policy with an average power constraint that is different from $\bar{P}$.

## 5. Deep Reinforcement Learning Based Power Control Policy

In this section, we describe the deep Q network (DQN) [11] based power control. First, we describe the DQN algorithm. We then propose a variant of DQN for constrained problems wherein we use a Lagrange multiplier to take care of the average power constraint. We use a multi-time scale stochastic gradient descent approach to also learn the Lagrange multiplier to obtain the right average power constraint. Finally, we change the learning step size from decreasing to a constant so that the optimal power control can track the time varying system statistics.

### 5.1. Deep Q Networks

DQN is a popular deep reinforcement learning algorithm to handle large state-space MDPs with unknown/complex dynamics $P(S_{t+1}|S_{t},P_{t})$. The DQN is a value iteration based method, where the action–value function is approximated by a neural network. Though there are several follow-up works providing improvements over this algorithm [17,48], we use this algorithm owing to its simplicity. We show that DQN itself is able to provide us with the optimal solution and tracking. These improvements may further improve the performance in terms of sample efficiency, estimator variance, etc. Earlier attempts for combining nonlinear function approximators, such as neural networks and RL, were unsuccessful due to instabilities caused by (1) correlated training samples, (2) a drastic change in policy with small change in function approximation, and (3) correlation between the training function and approximated function [10]. The success of DQN is attributed to addressing these issues with two key ingredients of the algorithm: experience replay memory M and target network, $Q_{\theta^{*}}$. The replay memory stores the transitions of an MDP, specifically the tuple $\left(S_{t},P_{t},r_{t},S_{t+1}\right)$. The algorithm then samples, uniformly, a random minibatch of transitions from the memory. This removes correlation between the data and smooths the data distribution change with the iteration. The algorithm has another neural network, approximating the value function, $Q_{\theta }$. The target network and randomly sampled mini-batch from the memory M form the training set for training the $Q_{\theta }$ at every epoch. This random sampling provides i.i.d samples for performing stochastic gradient descent with loss as follows:

(13) $$L_{Q}^{\pi_{\theta }}=\frac{1}{n}\sum_{j=1}^{n}{\left(Y_{j}-Q_{\theta }\left(S_{j},A_{j}\right)\right)}^{2}$$

where $Y_{i}=r_{i}+\gamma \;\max_{a^{\prime }}Q_{\theta^{*}}\left(S_{i},a^{\prime }\right))$. The iterations $\left\{\theta_{t}\right\}$ are given by the following:

(14) $$\theta_{t+1}\leftarrow \theta_{t}-\eta_{1}\left(t\right)\nabla_{\theta }L_{Q}^{\pi_{\theta }},$$

where $\eta_{1}\left(t\right)$, the step size, satisfies the following:

(15) $$\sum_{t=0}^{\infty }\eta_{1}\left(t\right)=\infty ,\;\sum_{t=0}^{\infty }\eta_{1}^{2}\left(t\right)<\infty ,\;\eta_{1}\left(t\right)\geq 0.$$

The weights of the target network $Q^{*}$ are held constant for $T_{target}$ epochs, thereby controlling any drastic change in policy and reducing correlation between *Q* and $Q^{*}$. This can be seen as a risk minimization problem in non-parametric regression with regression function $Q_{\theta^{*}}$ and risk $L_{Q}^{\pi_{\theta_{t}}}$. Readers are referred to [16] for an elaborate analysis of DQN. Theorem 4.4 in [16] provides a proof of the convergence and rate of convergence, using non-parametric regression bounds, when sparse ReLU networks are used, under certain smoothness assumptions on the reward function and the dynamics.

### 5.2. Adaptive Constrained DQN (AC-DQN)

The DQN algorithm is meant for unconstrained optimization. Since our problem has an average power constraint of $\bar{P}$, we consider the instantaneous reward in (12) with a Lagrange multiplier β. The long-term constraint depends on the Lagrange multiplier and can be quite sensitive to it. Thus, we design our algorithm, AC-DQN, to learn the appropriate β. We will see later, that this enables us to further modify our algorithm to track the changing statistics of the channel gains and arrival statistics. The AC-DQN algorithm is given in Algorithm 2. Here, we use the multi-timescale SGD as in [9]. In this approach, in addition to the SGD on $Q_{\theta }$, using a minibatch, we use a stochastic gradient descent on the Lagrange constant, β, as follows:

(16) $$\beta_{t+1}\leftarrow \beta_{t}+\eta_{2}\left(t\right)\nabla_{\beta }L_{P}^{\pi_{\theta }},$$

where $\nabla_{\beta }L_{P}^{\pi_{\theta }}=C_{P}\left(S_{t}\right)-\bar{P}$. Since the expectation in (11) is not available to us, we take $C_{P}\left(S_{t}\right)=\sum_{i=t-T_{W}}^{t}P_{i}\left(S_{i}\right)/T_{W}$, where $T_{W}$ is the finite horizon window. Additionally, $\eta_{1}$ and $\eta_{2}$ are required to follow the following [9]:

(17) $$\sum_{i=1}^{\infty }\eta_{1}\left(i\right)=\sum_{i=1}^{\infty }\eta_{2}\left(i\right)=\infty ,\;\sum_{i=1}^{\infty }\eta_{1}^{2}\left(i\right)+\eta_{2}^{2}\left(i\right)<\infty ,\;\frac{\eta_{2}\left(i\right)}{\eta_{1}\left(i\right)}\to 0.$$

**Tracking with AC-DQN:** The tracking of system statistics is essential to achieve optimal power control in a non-stationary system. In multi-time scale stochastic gradient descent, such as AC-DQN, step sizes $\eta_{1}\left(t\right)$ and $\eta_{2}\left(t\right)$ can be fixed to enable tracking. If $\eta_{2}<<\eta_{1}$, then the Lagrange multiplier changes much more slowly than the *Q*-function. Then, the two timescale theory (see, e.g., [9]) allows the Lagrange multiplier to adapt slowly to the changing system statistics but at the same time provide average power control. The solution reaches in a neighborhood of the optimal point. Although the convergence of this modified algorithm is not proved yet (even for the unconstrained DQN, convergence was proved only recently in [16]), our simulations show that the resulting algorithm tracks the optimal solution in the time varying scenario.

The time varying scenario in our setup results due to change in the request arrival statistics from the users and the changing channel gain statistics due to the motion of the users.
**Algorithm 2** Adaptive constrained DQN (AC-DQN) algorithm
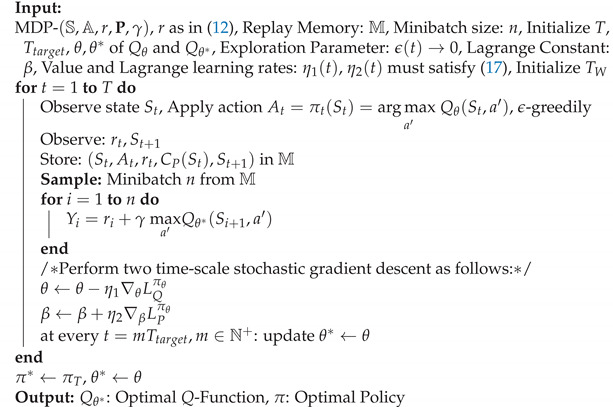


## 6. Integrated DSGD and AC-DQN (IDA)

We are now familiar with how the multi-time scale stochastic gradient descent can be used for optimization of a stochastic system with multiple objectives. We extend this idea to learn the optimal queuing strategy while learning the optimal power control policy and simultaneously satisfying the average power constraint. Toward this, we add DSGD as a third timescale to AC-DQN. Though DSGD internally has two stochastic gradient descent steps, we consider it to be a combined third step of IDA for conceptual clarity. We present our integrated DSGD and AC-DQN (IDA) in Algorithm 3. There are four learning rates involved in the algorithm. The four learning rates should satisfy the following criteria for convergence of the algorithm [9]:

(18) $$\begin{array}{cc} \sum_{i=1}^{\infty }\eta_{j}\left(i\right) & =\infty ,\;j=1,2,3,4,\\ \;\sum_{i=1}^{\infty }\sum_{j=1}^{4}\eta_{j}^{2}\left(i\right)<\infty & ,\;\frac{\eta_{j+1}\left(i\right)}{\eta_{j}\left(i\right)}\to 0,\;j=1,2,3. \end{array}$$

Though this criterion is required for convergence, we have seen that constant step sizes are helpful in tracking. So, we see our simulations with $\eta_{1}>\eta_{2}>\eta_{3}/T_{approx}>\eta_{4}/T_{approx}$.

Note 3: IDA can be used in systems with multiple objectives, e.g., a wireless network with cross layer objectives. It is important to select carefully the objective to be optimized in the slower timescale and in the faster timescale. In our setup, we run the learning steps for queuing policy (DSGD step) in a slower time scale to avoid drastic changes in the underlying MDP (of AC-DQN step).

Note 4: Step sizes in the algorithm are important hyperparameters. A good set of step sizes ensures a balance between speed and stability of the gradient descent steps. The choice is problem dependent and heuristical.

We now present the simulation results of all the algorithms presented in this paper.
**Algorithm 3** Integrated DSGD and AC-DQN algorithm (IDA)
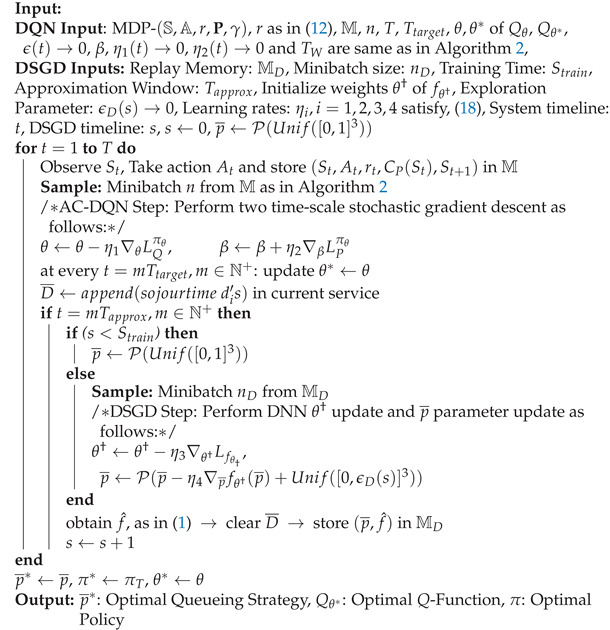


## 7. Simulation Results and Discussion

In this section, we first present the simulation results for our DSGD algorithm. We run the multicast system with constant transmit power. We compare the performance of our DSGD queuing algorithm against each queuing strategy proposed in [8]. Next, we compare the performance of AC-DQN and MADS power control policies. We show that the deep learning algorithm, AC-DQN, indeed achieves the global optimum obtained by the MADS algorithm, but unlike MADS, is also scalable with the system size (number of users). We further demonstrate that the AC-DQN algorithm tracks the changing system dynamics and obtains the optimal policy, adaptively. Finally, we consider IDA. We show, numerically, that the algorithm achieves the optimal point obtained by both DSGD and AC-DQN. We use Python 3.8 with the Tensorflow 2.4.0/Keras package for system implementation (the system and algorithm codes are available at https://github.com/rkraghu88/SchedulingPC_IDA, accessed on 12 October 2021).

### 7.1. Simulation Parameters

We consider three systems with varying system configurations as follows.

#### 7.1.1. Small User Case

Number of users L=4, catalog size M=100, file size F=10 MB, transmission rate C=10 MB/s, bandwidth B=10 MHz, channel gains ∼Uniform([0.1 0.2 0.3]) for two users with bad channel statistics and ∼Uniform([0.7 0.8 0.9]) for two users with good channel statistics. File popularity: uniform, (Zipf exponent = 0), average power constraint $\bar{P}=7$, simulation time = $10^{5}$ multicast transmissions.

#### 7.1.2. Moderate User Case

System parameters: power transmit levels = 20 (1 to 50), L=10, M=100, F=10 MB, C=10 MB/s, channel gains: exponentially distributed (∼exp(0.1) for bad channel, ∼exp(1.0) for good channel), R=10 MB/s, B=10 MHz, $\bar{P}=7$. File popularity: Zipf distribution with Zipf exponent = 1. Simulation time: $10^{5}$ multicast transmissions. In both the cases, we set the noise power as $N_{g}=1$.

#### 7.1.3. Large User Case

System Parameters: Same as Section 7.1.2 except, L=20.

#### 7.1.4. Hyperparameters

For DSGD, we consider a fully connected neural network with two hidden layers. The first layer has 32 nodes, and the second layer has 16 nodes. All layers have a ReLU activation function. $M_{D}=1000$, Minibatch size: $n_{D}=50$, $T_{train}=100$: Training Time, $S_{approx}=100$: Approximation Window, Initialize weights θ of $f_{\theta }$, ϵ(t)→0, $\eta_{1}\left(t\right)=0.01/\left(1+0.00001t\right)$, $\eta_{2}\left(t\right)=0.001/\left(1+0.00001t\left(llog\left(t\right)\right)\right)$.

In AC-DQN, we consider fully connected neural networks with two hidden layers for all the function approximations considered in the algorithms. Input layer nodes are assumed to be 2L and the output layer has 20 nodes, the number of transmit power levels. Each output represents the Q value for a particular action. The action space is restricted to be finite, as DQN converges only with finite action spaces. We use two hidden layers for the neural network, with 128 and 64 nodes, and the ReLU activation function is chosen. The other parameters are as follows: replay memory size |M|= 30,000, γ=0.9, $\epsilon_{0}=1.0$, $\epsilon_{decay}=0.98$, $\epsilon_{t}=\epsilon_{0}{\left(0.98\right)}^{t}$, $\eta_{1}=0.001$, $\eta_{1}^{decay}=0.00001$, $\eta_{2}=0.0001$, $\eta_{2}^{decay}=0.00001$, minibatch size (n)=64, $T_{target}=100$, and $T_{W}=200$.

Finally, in the IDA algorithm, we combine the parameters of both DSGD and AC-DQN. The step sizes are, however, held constant with the value of each step size at t=0.

### 7.2. Optimal Queueing Using DSGD

We consider the moderate user system in Section 7.1.2 for demonstrating the performance of DSGD. We assume the widely accepted IRM traffic model with unity zipf popularity for the 100 different file requests arriving at 10 users. The server is endowed, in different simulation runs, with different queuing strategies. We compare our DSGD based queuing strategy at the server with the individual queuing strategies, mentioned in Section 2. The server transmits the files with constant transmit power $\bar{P}=7$. We model the wireless fading to follow Rayleigh distribution. This introduces the errors in file transmissions.

**Figure 2:**
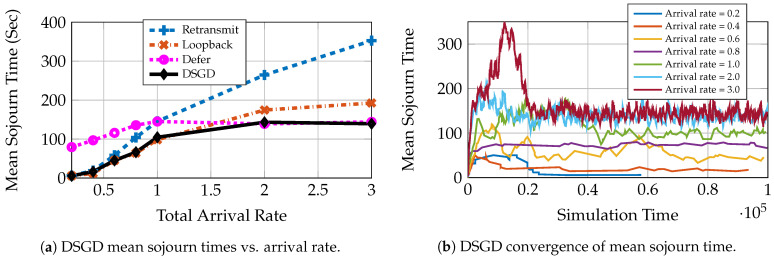
DSGD performance in parameterized multicast system with constant power policy, L=10, $\bar{P}=7$, Zipf Popularity (Zipf exponent = 1), Rayleigh fading with mean, 0.1 and 1.0 for bad and good users, respectively.

**Figure 3:**
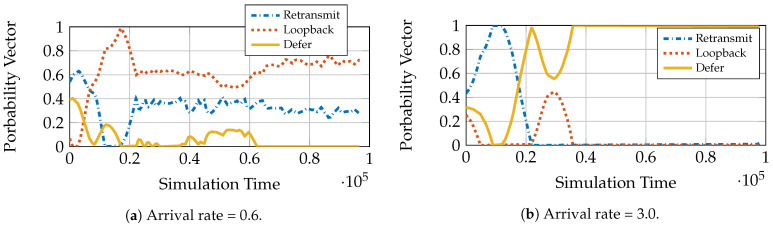
Probability convergence for L=10, $\bar{P}=7$, Zipf popularity (Zipf exponent = 1), Rayleigh fading with mean, 0.1 and 1.0 for bad and good users, respectively.

We see in Figure 2a that different queuing strategies are optimal at different rates for a constant transmit power $\bar{P}=7$ under fading. This is the typical case in practical systems. Depending on the request load, the system might need to adapt the queuing and service strategy. DSGD does precisely this. We can see in Figure 2b that the algorithm converges to the optimal mean sojourn time for the given power policy. We use a constant transmit power policy. Epochs 0 to $10^{4}$ are the initial training phase, and the algorithm starts learning thereafter and eventually converges. The policies chosen by the algorithm for arrival rates 0.6 and 3.0 are given in Figure 3a,b, respectively. We see that for rate 3.0, the algorithm converges to the defer strategy since it has the lowest mean sojourn time for this rate (Figure 2a). For rate 0.6 however, we see that DSGD gives a mixed policy with positive probabilities to retransmit and loopback and zero probability to defer. This is because both retransmit and loopback have the same mean delay performance, and the defer strategy performs poorly. This is the case where more than one optimal solution may be available, and the algorithm may converge to one or oscillate between different optimal points as neural network training progresses. The simulations show that the DSGD algorithm adaptively chooses the best among the three queuing policies or an equivalent mixed policy for different system statistics (arrival rates).

### 7.3. Optimal Power Control (AC-DQN vs. MADS)

We use the system setting of the small user case, specified in Section 7.1.1, since running MADS for a higher number of users is computationally prohibitive. We use the uniform popularity profile for the file requests. We also use uniform distribution for fading. This is just for the convenience of the calculations of state probabilities, $\left\{q_{k}\right\}$, in MADS as done in [8]. We compare the performance of AC-DQN and MADS for this system. We demonstrate our algorithm with more realistic distribution in the next section.

**Figure 4:**
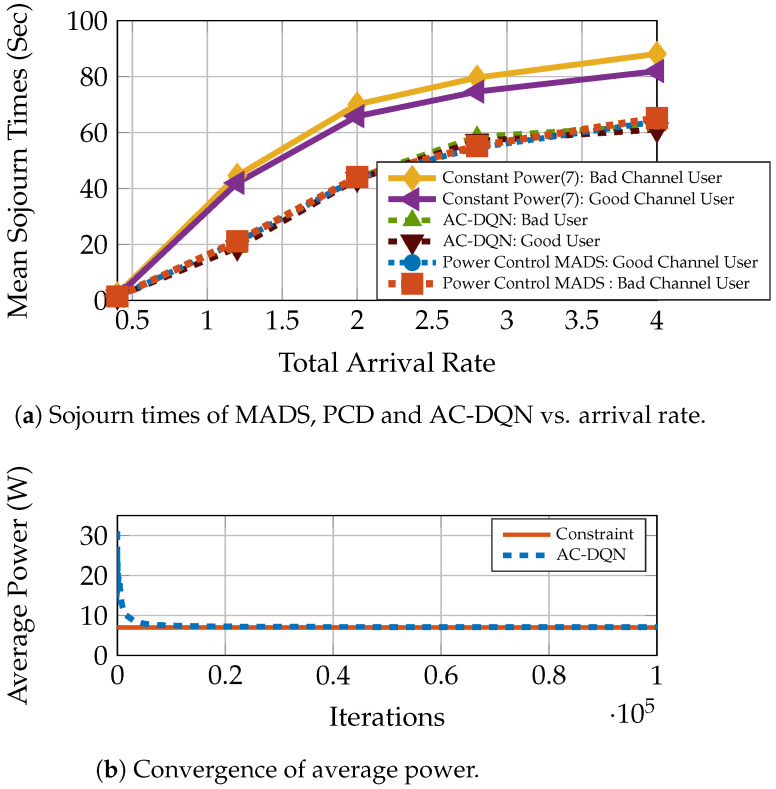
AC-DQN performance in 1-LB system with L=4, $\bar{P}=7$, uniform popularity, uniform fading.

We use the loopback queuing strategy for demonstrating AC-DQN. We see in subsequent sections that AC-DQN works even for other queuing strategies. To show the advantage of power control, we split the users in two equal sized groups, where one group has good channel statistics and the other bad channel statistics. We compare the performance of both the power control policies with the constant power control policy, where the transmit power $P_{t}$ is fixed to $P_{t}=\bar{P}$. Figure 4a shows a comparison of the mean sojourn times of constant power policy, $P_{t}=\bar{P}$, MADS and AC-DQN. We see from Figure 4a that AC-DQN achieves the same mean sojourn time as that by MADS but is much better than the constant power policy. Additionally, from Figure 4b, we see that AC-DQN satisfies the average power constraint.

### 7.4. AC-DQN Tracking Simulations in a Scaled Network

**Figure 5:**
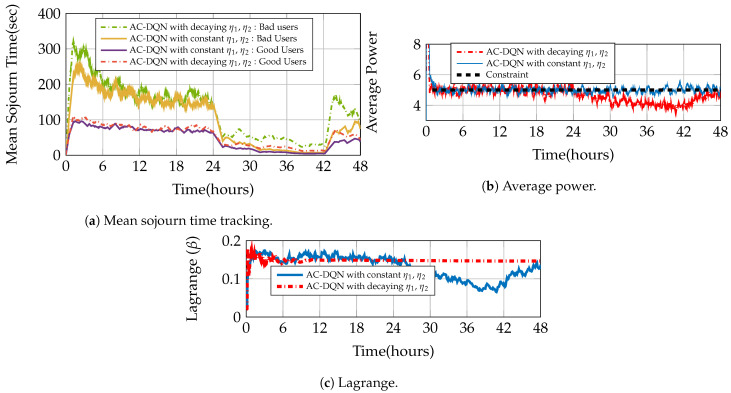
AC-DQN tracking performance in 1-LB system with with decaying vs. constant step sizes L=20, $\bar{P}=5$, Zipf(1) popularity, Rayleigh fading.

The AC-DQN provides similar improvements over the constant power scheme as above, even for a large user case [49] with the 1-LB queuing scheme. In this section, we show via simulations the tracking capabilities of AC-DQN for the large user case (Section 7.1.3). We demonstrate the importance of constant step sizes for $\eta_{1}$ and $\eta_{2}$, and the inability of decaying step sizes to track the changing system statistics. We consider a system where the arrival rates change over a period of 48 h. We fix λ=1.0 for the first 24 h. To make the learning harder for our algorithm, we change the rates abruptly every 6 h for the next 24 h as λ=0.6,0.5,0.4,0.8. This change in time period is just to illustrate the tracking ability in a more emphatic manner. This also captures the real-world scenario, where the request traffic to the base station varies with the time of the day. We fix $\bar{P}=5$. We calculate the mean sojourn time and average power, using a moving average window of 1000 samples in size. We run the AC-DQN algorithm for this system with (1) decaying $\eta_{1}$ and $\eta_{2}$ satisfying (17) and (2) constant step sizes, $\eta_{1}=0.001$ and $\eta_{2}=0.00003$. The rest of the parameters remain the same as in the large user case. We see in Figure 5a that the AC-DQN with a constant step size almost always outperforms the decaying step size. Specifically, after the first 24 h, the delay reduction is nearly 50 percent for a constant step size. The reason for this is evident from Figure 5b,c. We see in Figure 5c that the AC-DQN with a constant step size learns the Lagrange constant throughout the simulation time, whereas the AC-DQN decaying step size is unable to learn the Lagrange constant after the first 24 h. As can be seen in Figure 5b, this affects the average power achieved by the AC-DQN with a decaying step size. While a constant step size maintains the average power constraint of $\bar{P}=5$, the average power achieved by the decaying step size AC-DQN drops to 4. Hence, the decaying step size AC-DQN suffers suboptimal utilization of the available power. Thus, in practical systems, only constant step-size AC-DQN is capable of adapting to the changing system statistics. The effect of fixing the learning rates is seen in the small oscillations of average power around $\bar{P}=5$ in Figure 5b. This is the oscillation in a small neighborhood around the optimal average power. The smaller the step size, the lesser the oscillations.

### 7.5. Integrated Optimal Queueing and Power Control Using IDA

**Figure 6:**
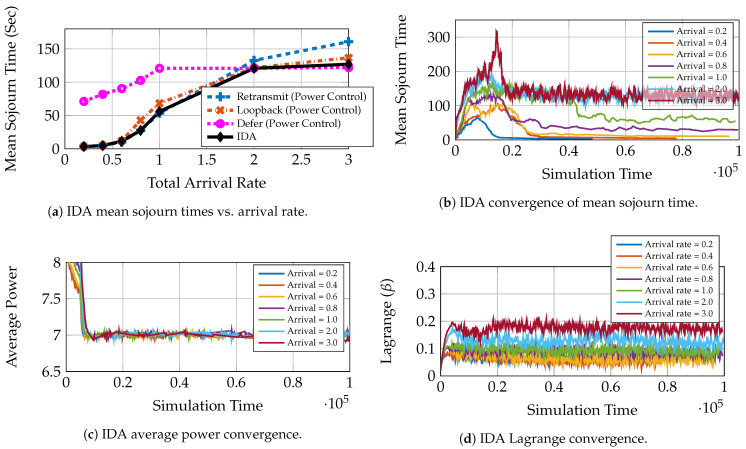
IDA Performance in parameterized multicast system with L=10, $\bar{P}=7$, Zipf popularity (Zipf exponent = 1), Rayleigh fading with mean, 0.1 and 1.0 for bad and good users, respectively.

**Figure 7:**
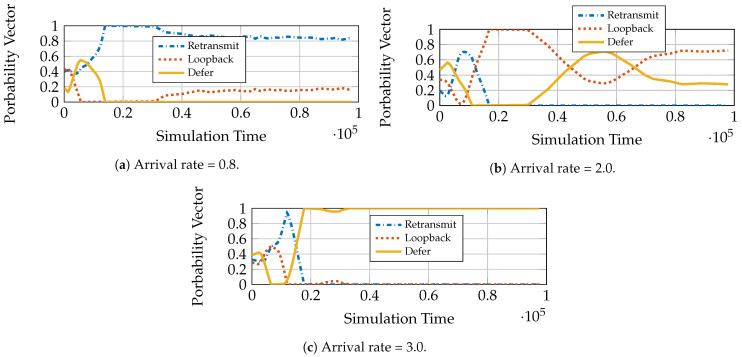
IDA convergence of queuing strategies for different arrival rates. L=10, $\bar{P}=7$, Zipf popularity (Zipf exponent = 1), Rayleigh fading with mean, 0.1 and 1.0 for bad and good users, respectively.

We have already seen the performance of power control for 1-LB (loopback case) for a large user system. In this section, we compare the performance of AC-DQN for different queuing strategies versus the IDA performance for the moderate user case (Section 7.1.2). We use Zipf popularity and Rayleigh fading for system simulation. First, in Figure 6a, we make an observation that AC-DQN drastically improves the mean delay performance for all the strategies as compared to the constant power policy in Figure 2a. We see that our IDA algorithm is able to choose a better strategy than the baselines in terms of the mean sojourn time. The convergence of the mean sojourn time for rates 0.2 to 3.0 is shown in Figure 6b. The more important capability of this algorithm is that it converges to a better mean sojourn time while maintaining the average power constraint. Figure 6c shows the convergence of the average power to $\bar{P}=7$ for all the rates. This is achieved by simultaneously controlling the Lagrange variable as seen in Figure 6d. A few interesting plots showing the convergence of probabilities for rates 0.8,2.0 and 3.0 are shown in Figure 7a–c, respectively.

We see, from Figure 7a, that for arrival rate 0.8, the queuing policy converges to a mixed policy with 0.8 probability assigned to retransmit and 0.2 assigned to loopback. This policy has the same optimal mean sojourn time as achieved by the best policy, retransmit, in Figure 6a. Thus, IDA gives additional optimal points for the algorithm to choose from. From Figure 6a we see that for rate 0.2, both defer and loopback have the same performance as AC-DQN. For arrival rate 3.0 (Figure 7c), however, IDA unambiguously chooses defer as the policy since it has the lowest mean sojourn time among the baselines in Figure 6a.

### 7.6. Tracking of User and Rate Variation Using IDA

In wireless content-centric networks, such as Netflix over 5G networks, the traffic is generally seen to start peaking in late afternoon and reach the maximum in the evening [50]. We show that power and queuing policies are tracked simultaneously via IDA in such a non-stationary setting.

**Figure 8:**
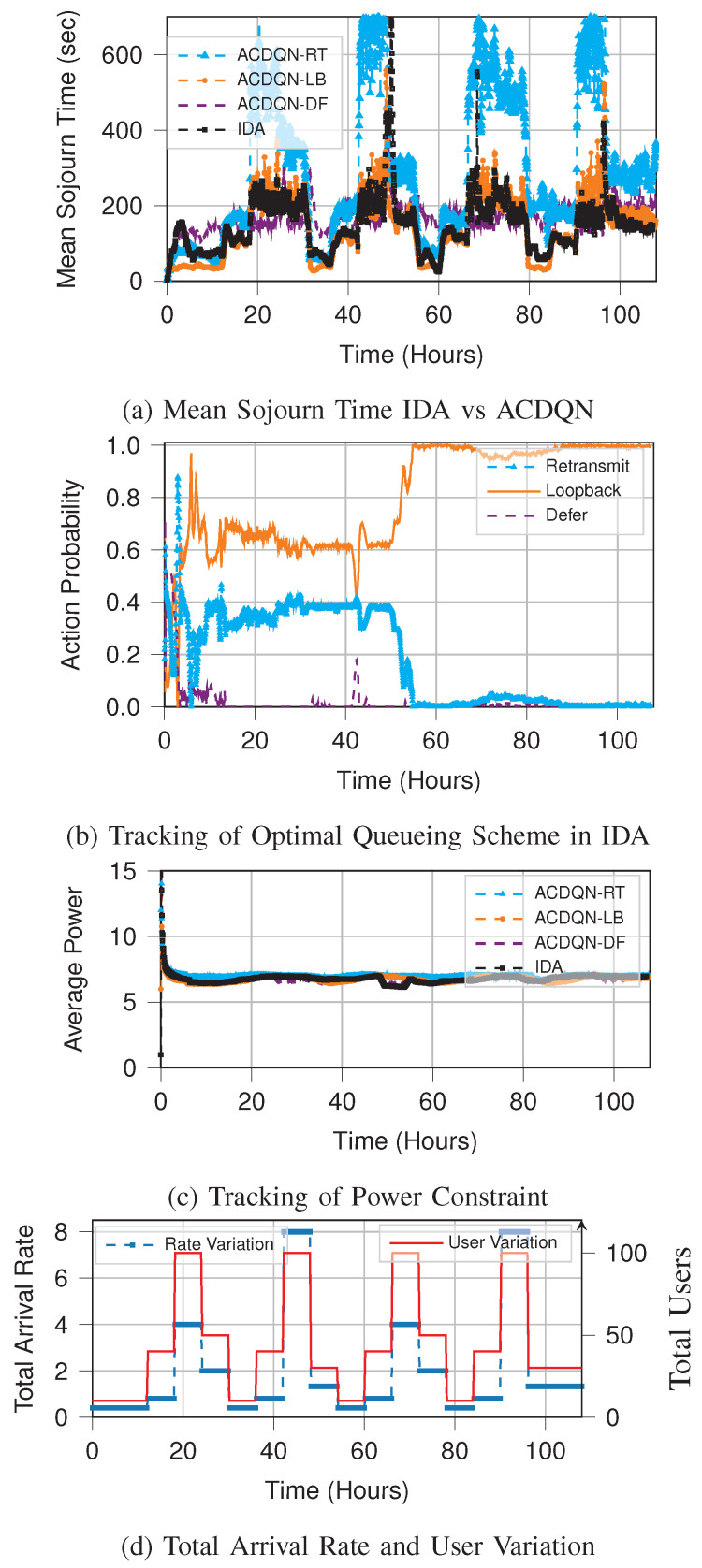
Tracking of optimal performance by IDA in a non-stationary environment, where total arrival rate to the base station/server varies over time.

We consider a system with a maximum of 100 users accessing a library of 100 files. The rest of the system parameters are as described in Section 7.1.2. The total user and rate variation mimics the real traffic as observed in [50]. The traffic starts increasing in the late afternoon and peaks in the evening. The traffic variations over a period of four and a half days are shown in Figure 8d. Except in the first 12 hours, the number of users and the request rate vary every 6 hours. It is important to note that our state formulation in AC-DQN and IDA allows for user variation in the system. The input to the neural networks has to be chosen based on the maximum number of users of 100 that the system admits. Thus, the state-space dimension is 200.

Due to a larger state-space dimension, to improve the sample efficiency of the Q learning, we make the network deeper and increase the number of nodes [39]. We scale the neural network size to three hidden layers with 256, 128, 64 nodes, and Input:200, Output:20. For a non-stationary setting, $T_{approx}$ should be chosen appropriately. A large $T_{approx}$ gives a bad estimate of the mean sojourn time and a small $T_{approx}$ increases the variance. Thus, we take $T_{approx}=40$. Similarly, to hold only relevant samples in the memory, we reduce the replay memory $M_{D}$ to 100 from 1000. This $\left\{T_{approx},M_{D}\right\}$ corresponds to a memory of ∼1 h. Additionally, to keep the exploration perpetual in a non-stationary system, we fix $\epsilon_{D}$ to a value of 0.005.

Figure 8 compares the performance tracking of IDA with respect to AC-DQN for individual queuing schemes when the arrival rate varies over time. This demonstrates the practical applicability of IDA where the environment is non-stationary. Figure 8a shows how IDA tracks the optimal mean sojourn time as the environment changes. During the first 12 h, the IDA assigns high probability to the retransmit policy. Since the traffic is very low in first 12 h, the learning process is slow. However, as the traffic picks up in the next 12 h, learning of the queuing policy and the power control policy, is accelerated, and IDA gets closer to the optimum performance among the three queuing schemes at 18 h. From there on, the learned optimal policy and power control are tracked near optimally. This can be seen in Figure 8a,b at time intervals [18,24], [42,48], [66,72], [90,96] hours.

Quick adaptation of IDA to changing system statistics can be seen emphatically at 50 h. At 48 h, the mean sojourn time spikes up (Figure 8a), due to change in the system statistics (Figure 8d). We deduce that the stochastic policy learned until 48 h is not optimal for the statistics of the system immediately after 48 h. Thus the $f_{\theta }$ learned in the DSGD step starts changing rapidly after 50 h, due to the small size of the replay memory, $M_{D}$. This induces rapid change in the descent step of parameter *p*, leading to a drastically different policy. We also note that such a spike in the mean sojourn time occurs at times 68 h and 96 h. However, these spikes are not large enough to warrant a drastic policy change.

We have simulated an environment which has abrupt variations. In the real scenario, the changes are much smoother. The algorithm can only do better in such a scenario. We also observe in Figure 8c that the IDA meets the power constraint. It does so by learning the optimal Lagrange multiplier corresponding to the instantaneous environment.

It is also important to mention that IDA is a stochastic algorithm performing non-convex optimization of a non-stationary system. The frequency of IDA getting stuck at a poor local optimum can, however, be controlled by appropriately tuning the hyperparameters, such as the DNN size, activation, step size, type of exploration noise, etc.

### 7.7. Discussion

We see from the simulations that the novel deep learning techniques, such as DSGD and AC-DQN, can achieve optimal performance while providing scalability with system size. Our two-timescale approach, AC-DQN, extends DeepRL algorithms, such as DQN, to systems with constrained control. It can be extended to systems with multiple constraints also. In such systems, each constraint is associated with a Lagrange multiplier.

For a stationary system, it is enough that the step sizes satisfy multi-timescale criteria similar to (17) (see [9]). However, if AC-DQN is used in systems with changing system statistics, the step sizes are kept constant. Choosing the step sizes is a trade-off between the tolerance of the constraint and the required algorithmic agility to track the system changes.

We have also demonstrated how IDA achieves the optimal queuing strategy among the baselines while obtaining the power control for such complex multicast systems. It is shown that deep neural networks, when appropriately used, can provide scalable control for large wireless networks, achieving several cross-layer objectives. IDA also tracks the optimal performance in a large non-stationary system with varying number of users.

## 8. Conclusions

This paper has considered a multicast downlink in a single hop wireless network. Fading of different links to users causes significant reduction in the performance of the system. Appropriate change in the queuing policies and power control can mitigate most of the losses. However, simultaneously obtaining adaptive queuing and power control for large systems is computationally very hard. We first developed a novel DNN assisted stochastic gradient descent algorithm to achieve optimality of the system to provide a lower mean sojourn time in a parameterized multicast system. Next, we showed that, using deep reinforcement learning, we can obtain optimal power control, online, even when the system statistics are unknown. We used a recently developed version of Q learning, the deep Q network, to learn the *Q*-function of the system via function approximation. Furthermore, we modified the algorithm to satisfy our constraints and also to make the optimal policy track the time varying system statistics. Finally, we proposed a novel deep multi-time scale algorithm which achieves the cross-layer optimization of queuing and power control, simultaneously. We showed that IDA also performs well in a large system with a non-stationary environment.

One interesting extension of this work would be developing an algorithm that could potentially provide a better state-action-dependent queuing strategy. Another future work could possibly include the caches at the user nodes and learning the optimal caching policy along with the power control using DeepRL. Future works may also consider applying IDA to multiple-base-station scenarios for interference mitigation. Further, extension of the approach to a multi-agent approach with decentralized execution as in [51] is an important research direction.

## Data Availability

The system and algorithm codes are available at https://github.com/rkraghu88/SchedulingPC_IDA (accessed on 12 October 2021).
